# Development of the PEO Based Solid Polymer Electrolytes for All-Solid State Lithium Ion Batteries

**DOI:** 10.3390/polym10111237

**Published:** 2018-11-08

**Authors:** Yu Jiang, Xuemin Yan, Zhaofei Ma, Ping Mei, Wei Xiao, Qinliang You, Yan Zhang

**Affiliations:** 1 College of Chemistry and Environmental Engineering, Yangtze University, Jingzhou 434023, China; librajiangyu@163.com (Y.J.); libramzf@163.com (Z.M.); PingMei@126.com (P.M.); WeiXiao@126.com (W.X.); 2 Key Laboratory of Optoelectronic Chemical Materials and Devices, Ministry of Education, School of Chemical and Environmental Engineering, Jianghan University, Wuhan 430056, China; QinliangYou@126.com

**Keywords:** solid polymer electrolytes, PEO, all-solid lithium ion batteries, functional polymers

## Abstract

Solid polymer electrolytes (SPEs) have attracted considerable attention due to the rapid development of the need for more safety and powerful lithium ion batteries. The prime requirements of solid polymer electrolytes are high ion conductivity, low glass transition temperature, excellent solubility to the conductive lithium salt, and good interface stability against Li anode, which makes PEO and its derivatives potential candidate polymer matrixes. This review mainly encompasses on the synthetic development of PEO-based SPEs (PSPEs), and the potential application of the resulting PSPEs for high performance, all-solid-state lithium ion batteries.

## 1. Introduction

Lithium-ion batteries (LIBs), as the most significant candidates for energy storage devices, have quickly occupied the global electrical consumer market due to their relatively high energy density, advanced operating voltage, and lack of a memory effect [1,2,3,4]. However, conventional LIBs with organic liquid electrolytes (OLEs) possess some inherent drawbacks, such as flammability, leakage, and environmental toxicity, which hinder their application in Electric Vehicles (EVs) or air-planes who need energy storage devices with high energy densities and, more importantly, high safety [5,6,7,8]. In this regard, replacing OLEs with all solid electrolytes (ASEs) seems to be a reliable solution for the aforementioned safety issues [9,10]. Compared with OLEs, ASEs have outstanding advantages in terms of mechanical strength, thermal tolerance, and electrochemical stability. Furthermore, ASEs can act as a separator to prevent electron transport while conducting lithium ions, which greatly simplifies the battery construction process, and also raises the possibility of using lithium metal as an anode for high energy density LIBs [11,12]. All in all, the application of ASEs in LIBs also has the following advantages: (1) The safety of the LIBs has been highly improved thanks to the absence of an organic liquid electrolyte. (2) The high thermal stability of ASEs makes the casing module and cooling system more simplified, which can reduce the weight of the battery, thereby increasing the energy density. (3) A plurality of electrodes can be stacked in series in one unit due to the solid state characteristics, which makes it possible to prepare large voltage single batteries. (4) Wide electrochemical window (up to 5 V or more) of the ASEs makes it possible to use high voltage electrode materials, thus improving the operating voltage [13,14,15,16]. Furthermore, the cost of the LIBs can be significantly reduced by replacing the liquid electrolyte with SPEs. First, the fabrication cost of SPEs is much lower than that of liquid electrolyte-separator systems; second, the use of positive temperature coefficient resistors, fuses, and solid packages with superior mechanical strength is unnecessary in SPEs-based LIBs, attributing to high temperature tolerance and advanced the mechanical strength, thereby saving production costs.

According to the classification of components, ASEs can be divided into inorganic solid electrolytes and organic solid electrolytes. Inorganic solid electrolytes (such as NASICON Li–Al–Ti–PO_4_) possess high ion conductivity at room temperature (can reach more than 10^−4^ S·cm^−1^ at 25 °C) [17,18]. Although inorganic solid electrolytes have superior ion conductivity and high Li^+^ transference numbers, their practical usage is still impeded by their defects, such as large grain boundary resistance, poor interface compatibility between inorganic solid electrolyte and electrode, relatively cumbersome fabrication process, and large energy barrier for lithium ion electrolyte migration in electrode interface. These features will cause a series of problems, especially the growth of lithium dendrites during the charging–discharging process [19,20,21]. Moreover, inorganic solid electrolytes are too hard and brittle for flexible battery applications. In contrast, polymer electrolytes (PEs) exhibit excellent interfacial compatibility with both electrodes, and favorable mechanical properties. Additionally, the energy density of LIBs can be greatly improved due to the usage of PEs which have a much lower density than those of inorganic solid electrolytes [22,23].

Polymer electrolytes can be generally divided into two kinds, depending on the state of the component, namely, gel polymer electrolytes (GPEs) and solid polymer electrolytes (SPEs). GPEs have been commercialized owing to their excellent ion conductivity which shows no significant decrease compared to that of the OLEs. The emergence of non-volatile polymer electrolytes means that LIBs exhibit more diversification in shape, adapting to the appearance variations of small electronic products [24,25]. However, GPEs still use a flammable organic liquid as the solvent of lithium salts, while SPEs solve lithium salts through interaction of their ether oxygen with no organic liquid solvent, which can significantly improve the high temperature tolerance of electrolytes. Furthermore, the SPEs show superior mechanical properties than GPEs since no organic liquid solvent is used, which can effectively suppress the growth of Li dendrites, thus avoiding internal short circuits and significantly increasing the safety property of batteries [19,26].

Nowadays, the mainstream polymer matrix of the SPEs is still PEO and its derivatives, resulting from its good chain flexibility, superior electrochemical stability to lithium metal, low glass transition temperature (*T*_g_), and excellent solubility with conductive lithium salts [27,28]. However, high crystallinity of PEO leads to low ion conductivity (10^−8^–10^−6^ S·cm^−1^) and inferior Li^+^ transference numbers (0.2–0.3) at room temperature, which seriously affects the high rate capability of LIBs. For this reason, various methods have been adopted to synthesize PSPEs with good mechanical properties, superior electrochemical stability, and especially, high ion conductivity [29,30].

## 2. Synthetic Development of PSPEs

### 2.1. Copolymerization

**Figure 1:**
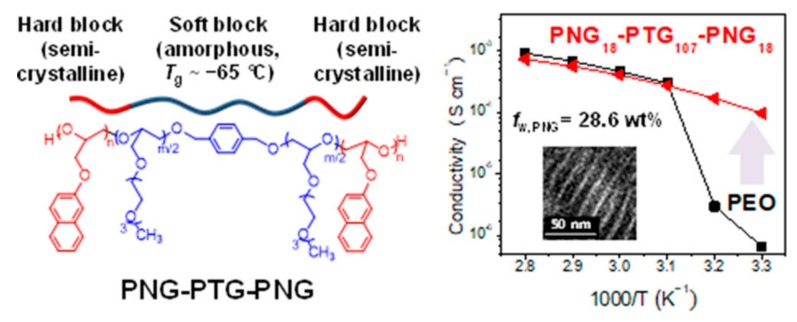
Chemical structure and ionic conductivity of PNG–PTG–PNG [38].

Copolymerization with another polymer can effectively decrease crystallinity and improve the amorphous area (considered as the main conductive area of Li^+^), thus increasing the ionic conductivity of PSPEs at room temperature [30,31]. A typical example is copolymerization of ethylene oxide and propylene oxide (PEO–PPO) (with a molecular weight of 10^5^), whose ionic conductivity can be as much as 2.6 × 10^−5^ S·cm^−1^ at the room temperature [32,33]. Other random copolymers like PEO–epichlorohydrin and methyleneoxy–PEO all exhibit excellent electrical conductivity due to the random copolymerization process, and show an enhancement of ion conductivity by increasing the EO ratio in the obtained random copolymer. Nishimoto et al. [34] synthesized poly[ethylene oxide-co-2-(2-methoxyethoxy)ethyl glycidyl ether] P(EO-MEEGE) as a high molecular weight (>10^6^) polyether comb polymer, which can form elastic polymer electrolyte films with electrolyte salts at room temperature and no chemically cross-linked structures. Compositing with lithium bis(trifluoromethylsulfonyl)imide (LiTFSI), the obtained polymer electrolytes with 9% (mole ratio) MEEGE exhibit high ionic conductivities, i.e., 10^−4^ S·cm^−1^ at 30 °C and 10^−3^ S·cm^−1^ at 80 °C, attributable to the synergistic effect of the decrease in crystallinity and the increase in the number of highly-mobile ether side chains. Z. Floriańczyk et al. [35] synthesized a PEO–PAAM copolymer by the thermopolymerization of high-molecular-weight PEO and methyl methacrylate (MMA). MMA chain grafting on the PEO chain can significantly reduce the *T*_g_ and crystallinity of PEO at room temperature. After compositing with LiClO_4_, the LiClO_4_–PEO–PAAM-based PSPEs show good mechanical properties. Moreover, the ion conductivity of the PSPEs can reach up to 6.6 × 10^−5^ S·cm^−1^ at room temperature, and 10^−3^ S·cm^−1^ at 100 °C. PAAM can also be a potential candidate to copolymerize with PEO, ascribed to the flexibility and the strong polarity which can promote the dissociation of lithium salt. The optimal room temperature ion conductivity of PEO–PAAM can reach up to 4 × 10^−5^ S·cm^−1^ [36]. Ayan Ghosh et al. prepared a LiBOB–PEO–b–(PMMA–ran–PAAMLi) solid polymer electrolyte membrane by copolymerization of PEO–PMMA block copolymer with lithium methacrylate (MAALi), followed by compositing with lithium bis(oxalate)borate (LiBOB). The obtained PSPEs show tremendous Li^+^ transference numbers, i.e., reaching up to 0.9 at room temperature [37]. Lee et al. synthesized triblock copolymers based on poly(ethylene oxide) (PEO), poly (2-naphthyl glycidyl ether)–block–poly [2-(2-(2-methoxyethoxy)ethoxy) ethyl glycidyl ether]-block-poly(2-naphthyl glycidyl ether)s (PNG–PTG–PNGs) shown in Figure 1, which maintained high ion conductivity at room temperature due to the low *T*_g_ (about −65 °C) and amorphous nature of the PTG blocks. It is worthy of note that the ion conductivity of the PNG_18_–PTG_107_–PNG_18_ can still reach 9.5 × 10^−5^ S·cm^−1^, even though the fraction of amorphous regions was reduced owing to the crystalline PNG domains resulting from the formation of excellent Li^+^ transport pathways driven by the microphase separation of PNG–PTG–PNGs [38]. Moreover, the degradation temperatures at 5 wt % loss values (*T*_d_, 5%) of PNG–PTG–PNGs were in the range 371−390 °C, implying that solid electrolytes prepared from PNG–PTG–PNGs have a broad range of operating temperatures.

### 2.2. Crosslinking

**Figure 2:**
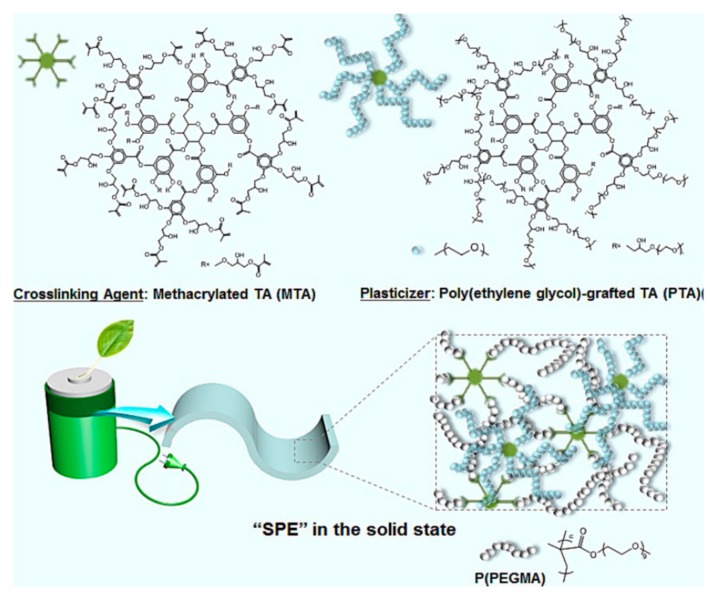
Conceptual illustration of solid polymer electrolytes comprising a crosslinking agent and plasticizer based on functionalized tannic acid [41].

Crosslinking is also an effective method to decrease crystallinity, while also substantially improving mechanical strength and thermal stability of the PSPEs. Matoba et al. [39] synthesized ethylene oxide copolymers containing lots of side chains and unsaturated bonds. After compositing with various lithium salts, the conductivity of the obtained PSPEs can reach up to 10^−5^–10^−4^ S·cm^−1^ at 30 °C, demonstrating that good ionic conductivity and strong mechanical properties can be obtained simultaneously through the optimization of crosslinking conditions. Jimin Shim et al. [40] fabricated PSPEs based on polysiloxane derivatives, having ion-conducting PEO side chains crosslinked with modified gallic acid. PSPEs exhibited extraordinary ionic conductivity (4.0 × 10^−4^ S·cm^−1^ at 60 °C) and mechanical strength thanks to the formation of highly-crosslinked polymer networks. Jong-Chan Lee et al. [41] reported a one-pot polymerization method to synthesize PSPEs (as shown in Figure 2) using poly(ethylene glycol) methyl ether methacrylate (PEGMA) as an ion-conducting monomeric unit and tannic acid (TA)-based crosslinking agent and plasticizer. According to testing results, the ionic conductivity value of PSPEs (~10^−4^ S·cm^−1^ at 30 °C) is one order of magnitude larger than that of linear P(PEGMA) in the waxy state. This superior ionic conductivity is attributed to the large number of crosslinking sites of methacrylated tannic acid and the addition of PEG grafted tannic acid as the plasticizer, which can increase the mobility of ion-conducting chains. The Td, 5% in the SPEs were found to be approximately 290 °C, indicating that MPEs can be used for high-temperature applications such as electric vehicles and energy storage systems. Guo et al. [42] developed a novel solid-state polymer electrolyte with an interpenetrating poly(ether-acrylate) (ipn-PEA) network, which was synthesized by photopolymerizing ion conductive polyethylene oxide (PEO) and branched acrylate. The obtained ipn-PEA electrolyte exhibited the bifunctionality of high ion conductivity (2.2 × 10^−4^ S·cm^−1^) at room temperature and high mechanical strength (ca. 12 GPa), resulting from the ideal combination of plasticity and rigidity inherited from PEO and PEA respectively. Therefore, the ipn-PEA-based, all-solid battery delivered an admirable specific capacity and cycling stability at 1 C rate at room temperature. Moreover, the ipn-PEA electrolyte showed a distinct effect on blocking Li dendrite growth. To enhance the mechanical strength of polymer electrolytes, Tong et al. [43] reported a novel, all-solid-state polymer electrolyte with an interpenetrating network prepared via a one pot synthetic strategy using a ring-opening polymerization technique. More importantly, the ionic conductivity of the polymer electrolytes could easily be optimized by varying the GMA content in the 3-arm prepolymer, the crosslinking density, or lithium salt concentration. The electrolyte possessed a high electrochemical stability window of 4.5 V, and had good stability to suppress Li dendrite growth in lithium metal batteries.

### 2.3. Hyperbranching 

**Figure 3:**
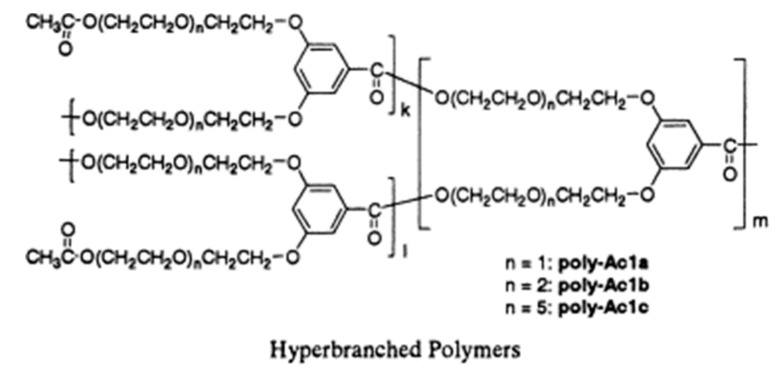
Chemical structure of hyperbranched polymers [46].

Nowadays, hyperbranched polymers have received extensive attention due to their unique molecular structures which can immensely improve the electrochemical performance of the PSPEs. Specifically, the highly branched structure can effectively decrease crystallinity, while the approximate spherical molecule configuration can tremendously increase the free volume of the polymer. Hawker et al. [44], for the first time, synthesized hyperbranched polymer electrolytes by using PEO as a raw material. The ion conductivity of the obtained hyperbranched polymer can reach up to 10^−5^ S·cm^−1^ due to the completely amorphous structure. Watanabe et al. [45] prepared a network polymer through the photocrosslinking of macromolecular monomers which contained hyperbranched polyether short chains. The obtained polymer electrolyte had the highest ionic conductivity (~10^−4^ S·cm^−1^, at 30 °C) when the molecular weight of macromolecular monomers was around 1000. Itoh et al. [46] synthesized hyperbranched polyphenyl formate containing PEO short chains (Figure 3). The ionic conductivity of the obtained PSPEs can reach 10^−7^–10^−6^ S·cm^−1^ at room temperature and nearly 10^−4^ S·cm^−1^ at 80 °C. Moreover, they demonstrated that ionic conductivity and Li^+^ transference number of the PSPEs can be increased simultaneously by adding poly[bis(triethylene glycol)benzoate]. 

### 2.4. Blending

Blending with other polymers has been widely used to increase the ionic conductivity of PSPEs, because it can suppress crystallization and improve *T*_g_ of PEO. To synthesize PSPEs, Przyluski blended PEO with polyacrylamide (PAM), and then composited it with LiClO_4_. The obtained PSPEs exhibit a superior ionic conductivity (10^−4^ S·cm^−1^) at room temperature [47]. Tanaka et al. [48] certified that the ionic conductivity of the PEO–LiClO_4_ electrolyte blending with polyaziridine (PEI) is three orders of magnitude higher than that of the pure PEO–LiClO_4_ electrolyte. The ionic conductivity of the obtained PEI–PEO–LiClO_4_ electrolyte can reach 10^−4^ at room temperature. This was confirmed by the sharp decrease of ionic conductivity of the PEO–LiClO_4_-based electrolyte caused by crystallization being efficiently suppressed. Although ionic conductivity can be significantly improved by blending, the mechanical properties of the PSPEs will be compromised. 

### 2.5. Composite Polymer Electrolyte

**Figure 4:**
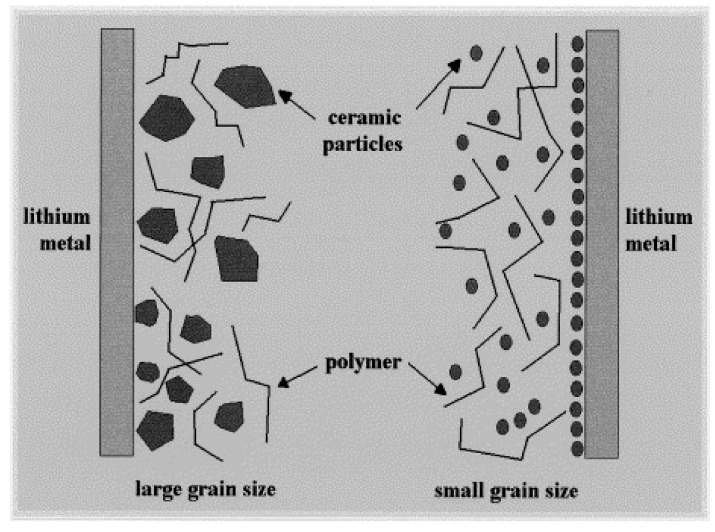
Schematic illustration of Metal lithium electrode/composite electrolyte interface [52].

Since there is a trade-off between ion conductivity and mechanical properties in PSPEs, plenty of additives have been selected to synthesize a perfect polymer electrolyte which exhibits good mechanical strength, superior electrochemical stability, and high ionic conductivity. In general, inorganic additives can improve the interface between the polymer electrolyte and the electrode, meanwhile improving the interfacial compatibility between the polymer electrolyte and the lithium electrode by capturing impurities in the battery system. Inorganic materials are generally divided into two categories [49,50,51]: those that are inactive in lithium ion conduction process, e.g., SiO_2_, TiO_2_, ZrO_2_, Al_2_O_3_, ZnO, MgO and etc., and those that are active in lithium ion transport, e.g., Li_3_N, LiAlO_2_, and so on. Scrosati et al. [52] studied the influence of different inorganic fillers (γ-LiAlO_2_, Al_2_O_3_, SiO_2_) on the performance of a solid-polymer-electrolyzed PEO–LiCF_3_SO_3_ system. It was found that the addition of inorganic fillers could not only effectively cover the electrolyte membrane, thereby improving the interface stability with the metal lithium electrode, but also highly enhance the ionic conductivity and lithium ion transport performance of the SPEs. A schematic diagram of the interface between the composite electrolyte and the metal lithium is shown in Figure 4. Inorganic fillers can adsorb liquid impurities in the battery system and form a stable passivation film layer on the surface of the metal lithium anode, thus achieving a long cycling life for LIBs.

**Figure 5:**
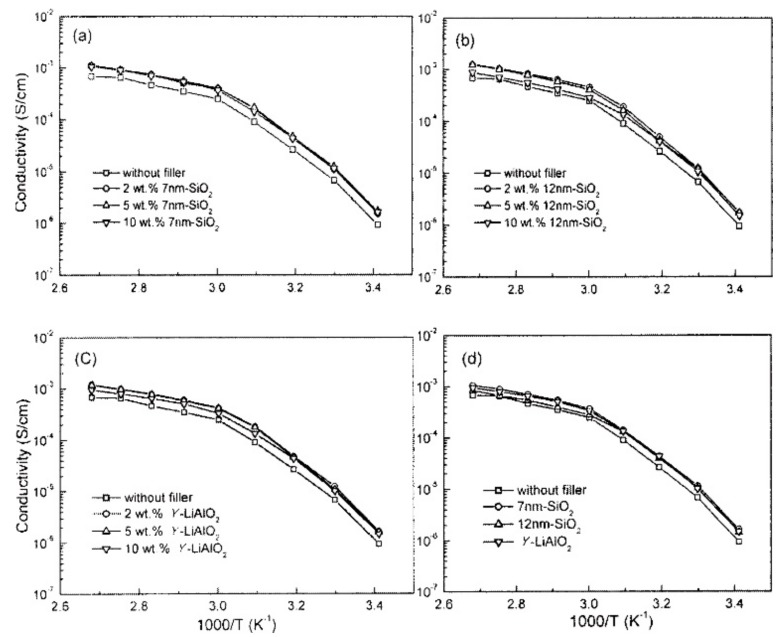
Arrhenius plots of the ionic conductivity of P(EO)_20_LiBETI + *x* (*x* = 0, 2, 5, and 10) wt % filler (**a**) 7 nm SiO_2_, (**b**) 12 nm SiO_2_, (**c**) γ-LiAlO_2_ and (**d**) P(EO)_20_LiBETI + 10 wt % filler polymer electrolyte sandwiched between Cu electrodes [53].

Passerini et al. studied the effect of different sized inorganic fillers (7 nm SiO_2_, 12 nm SiO_2_, microsize γ-LiAlO_2_) on lithium ion conduction performance in PEO_20_–LiBETI system [53]. The effect of different types and filler ratios on ionic conductivity is shown in Figure 5. They demonstrated that the addition of different types and proportions of fillers can increase the ionic conductivity of polymer electrolyte membranes with temperatures below the melting point. It is worthy of note that the polymer electrolyte membrane with same mass ratio of different inorganic fillers showed almost same ion conductivity below 90 °C, which also proved that the size of the inorganic fillers has little effect on the ionic conductivity of the electrolyte membrane within a certain range. The interaction between inorganic filler and polymer can be categorized as follows [51,54,55]: (1) The special functional group on the surface of the inorganic filler can form a hydrogen bond with the oxygen atom on the PEO segment which will force the linear polymer chain deformation, thereby reducing the crystallinity of the PEO polymer; (2) the inorganic fillers acted as a cross-linking center, interacting with both polymer segment and the lithium salts that will change the crystalline nature of the polymer segment and optimize the transport path for lithium ions. The binding competition between the inorganic fillers and the anion group of lithium salts can strongly promote the dissociation of lithium salt, thus improving the conductivity of lithium ions and Li^+^ transference number; (3) effective medium theory. Lithium ions can separately transport in polymer matrix, inorganic fillers, and the ionic conductor layer situated between polymer matrix and the inorganic fillers. This synergistic effect means that inorganic-PSPEs present excellent ion conductivity.

### 2.6. Ceramic-Polymer Solid Electrolyte

**Figure 6:**
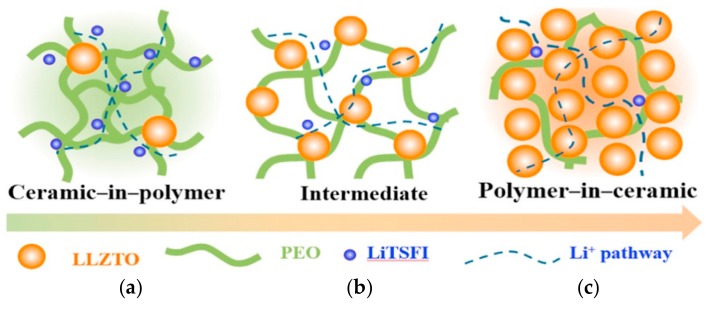
Schematic illustration for PEO–LLZTO ceramic/polymer solid electrolyte: (**a**) ceramic-in-polymer; (**b**) intermediate; (**c**) polymer-in-ceramic [61].

As described above, ceramic solid electrolytes have superior ionic conductivity (up to 10^−4^ S·cm^−1^ at 25 °C) and high Li^+^ transference number. However, their practical application has been limited by their inherent drawbacks, i.e., large grain boundary resistance and poor electrode–electrolyte interface compatibility. Compositing ceramic solid electrolytes with PSPEs, the obtained electrolytes can exhibit advanced ionic conductivity, high Li^+^ transference numbers, and favorable electrode electrolyte contact interfaces [56]. Zhao et al. added ternary sulfide solid electrolyte Li_10_GeP_2_S_12_ as a filler into the PEO_18_LiTFSI solid polymer electrolyte. The corresponding relationship between ionic conductivity and temperature was tested [57]. The best ion conductivity was observed when the Li_10_GeP_2_S_12_ filler ratio was at 1 wt % (1.18 × 10^−5^ S·cm^−1^ at 25 °C and 1.23 × 10^−3^ S·cm^−1^ at 80 °C, respectively). The stable electrochemical window of the Li_10_GeP_2_S_12_–PEO_18_LiTFSI composite electrolyte attained 5.7 V. The obtained electrolyte exhibited a good interfacial compatibility with a lithium cobaltate LiCoO_2_ and lithium iron phosphate LiFePO_4_ cathode. To synthesize high-electrochemical-performance PSPEs, Narin et al. [58] replaced the inactive inorganic filler with a solid electrolyte Li_1.3_Al_0.3_Ti_1.7_(PO_4_)_3_ (LATP). The ion conductivity of the obtained electrolyte could reach (1.9 ± 0.2) × 10^−4^ S·cm^−1^ at 40 °C with a mass ratio of the LATP up to 66 wt %. Wen et al. [59] prepared two NASICON-type inorganic solid electrolytes, i.e., Li_1.4_Al_0.4_Ti_1.6_(PO_4_)_3_ (LATP) and Li_1.5_Al_0.5_Ge_1.5_(PO_4_)_3_ (LAGP), and composited them with PEA_18_LiTFSI to fabricate an organic-inorganic composite solid electrolyte. The test results certified that the two obtained composite solid electrolytes had the best ionic conductivity at room temperature with a mass ratio of inorganic solid electrolyte up to seventy percent (LATP-PEO_18_LiTFSI 1.86 × 10^−4^ S·cm^−1^ and LAGP–PEO_18_LiTFSI 1.11 × 10^−4^ S·cm^−1^). Since lithium anodes are apt to react with Ti^4+^, the interfacial stability of LAGP–PEO_18_LiTFSI outperforms LATP–PEO_18_LiTFSI, as proven by cyclic voltammetry. To present the remarkable electrochemical performance of the LAGP–PEO_18_LiTFSI in practical applications, an all-solid-state lithium-ion battery (ternary cathode material/LAGP–PEO_18_LiTFSI/Li) was prepared, which exhibited excellent cyclicity. Kim et al. [60] prepared a Li_1.5_Al_0.5_Ge_1.5_(PO_4_)_3_–PEO_18_LiClO_4_ composite solid electrolyte which showed marvelous ion conductivity (above 2.6 × 10^−4^ S·cm^−1^ at 55 °C) with a mass ratio of Li_1.5_Al_0.5_Ge_1.5_(PO_4_)_3_ up to 60%, resulting from the high electrochemical stability of the composite electrolyte and the good interfacial contacts with electrodes. Goodenough et al. [61] synthesized an Organic–inorganic, composite solid electrolyte based on PEO containing garnet Li_6.4_La_3_Zr_1.4_Ta_0.6_O_12_ compositing with LiTFSI (PEO–LIZTO–LiTFSI). Depending on the mass ratio of the PEO matrix and the ceramic electrolyte filler, this composite electrolyte could be classified as ceramic-in-polymer or polymer-in-ceramic (Figure 6). The obtained electrolyte exhibited superior electrochemical performance, including a wide electrochemical stability window (up to 5 V), high ion conductivity (above 10^−4^ S·cm^−1^ at 55 °C), excellent capability to suppress Li dendrite growth, and enhanced interface stability against a Li anode. The assembled all-solid state battery (LiFePO_4_/PEO–LIZTO–LiTFSI/Li) showed marvelous cycling stability in a voltage range of 2.6–4.0 V at 0.2 C and 55 °C (a stable specific capacity of 127 mAh·g^−1^ after 50 cycles, with an ideal Coulombic efficiency of 100%).

### 2.7. Salt-Soluble Polymer Electrolyte

Although various modification methods have been applied, the ionic conductivity of the PSPEs still stays within the range of 10^−5^–10^−4^ S·cm^−1^ at room temperature, which is much lower than that required by practical applications (10^−3^ S·cm^−1^ at room temperature). It is generally considered that the inferior ionic conductivity is ascribed to the strong coupling of ionic motion and polymer segmental motion. To address these issues, lots of effort has been made to explore a whole new electrolyte without coupling of ionic motion and polymer segmental motion, for instance, salt-soluble polymer electrolytes (SSPEs). Unlike ordinary PEs, SSPEs are fabricated by dissolving polymers into lithium salts which determines their main properties. Owing to high ion concentrations, the ion-conducting mechanism of the SSPEs is similar to that of molten salt, as determined by the ion transportation and independent of the polymer segmental motion. The polymer mainly acts as a carrier in the electrolyte which will significantly strengthen the mechanical properties of SSPEs. Angel et al. reported the first SSPEs in 1993 [62], which were fabricated by adding a small amount of polyoxypropylene (PPO) into a mixture of lithium salts (such as LiI–LiOAc–LiClO_4_). The obtained SSPEs exhibited superior ionic conductivity, i.e., up to 10^−3^ S·cm^−1^ at the room temperature. Ohno et al. prepared molten-salt-type polymer brushes with different ethylene oxide (EO) unit numbers and different tethering structures. The highest ionic conductivity of the obtained PEO-based SSPEs can reach 1.49 × 10^−4^ S·cm^−1^ at 30 °C after used TFSI^-^ as the counter anion species of the polymer. They certified that PEO-tethering was an efficient way to suppress the drop of ionic conductivity of the molten salts after polymerization, and confirmed that the ionic conductivity of the PEO-based SSPEs improved with an increase of ethylene unit number [63].

### 2.8. Polymeric Single Ion Conductor

**Figure 7:**
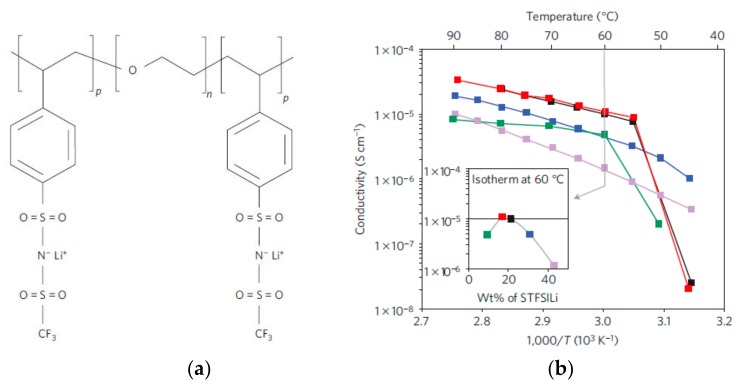
(**a**) Chemical structure of the single-ion conductor triblockcopolymer P(STFSILi)–b–PEO–b–P(STFSILi); (**b**) Conductivity performances. Plots of conductivity as a function of inverse temperature for P(STFSILi)–PEO–P(STFSILi) [66].

Normally, the cations and anions of the electrolyte will accumulate at the anode and cathode when an electric potential is applied, resulting in reverse polarization which will seriously jeopardize rate performance of LIBs. This effect will be reduced by covalently bonding the anion to the polymer chain, namely, a single ion conductor. Due to the immobilizing of the anion, a single ion conductor presents a high lithium transport number. Besides that, the single ion conductor can also inhibit the reaction between the anion and electrode, which facilitates a reduction in the passivation layer on the electrode surface, thus enlarging the operating voltage window of the battery [27,64,65]. To simultaneously enhance ionic conductivity, lithium transport number, and mechanical properties, Bouchet et al. designed a new single-ion polymer electrolyte based on self-assembled polyanionic BAB triblockcopolymers P(STFSILi)–PEO–P(STFSILi), as seen in Figure 7, where the B block was based on poly(styrene trifluoromethanesulphonylimide of lithium) P(STFSILi) and the central A block was based on a linear poly(ethylene oxide) (PEO) [66]. The obtained electrolyte showed remarkable mechanical strength with single-ion conductivity (1.3 × 10^−5^ S·cm^−1^ at 60 °C) almost 5 times higher than that of other state-of-the-art materials. Due to the superior electrochemical stability, the electrochemical window of the electrolyte is extended to up to 5 V versus Li^+^/Li, thereby improving the energy density of the battery. Although single ion conductors can greatly improve the electrochemical performance of the PSPEs, the ion conduction mechanism for single ion conductors remains unclear. Maranas [67] studied a single ion conductor possessing a sulfonate anion (SO^3−^) covalently bonded to a PEO backbone, and attempted to identify the key elements that contributed to high conductivity in single ion conductors. They confirmed that the morphology of the samples, especially the ion aggregation, played an important role in ion conduction for single ion conductors.

## 3. Conclusions

Safety issues of battery combustion and explosions due to flammable solvents remain a major impediment to the practical application of OLEs in EVs, drones, and airplanes. PSPEs are a promising candidate given their outstanding safety and electrochemical performance, thanks to their from all solid state and the absence of any organic liquid solvent, flexibility, superior electrochemical stability to lithium metal, excellent solubility to the conductive lithium salt, and relatively low *T*_g_. However, the poor ionic conductivity and the mechanical strength of PSPEs hinder rate performance and the cycling life of the battery. To address this problem, many strategies have been employed, and some progress has been made. The electrochemical performance of modified PSPEs is still unsatisfactory, and rate capability and cycling stability cannot meet the requirements of commercially-used LIBs. Nevertheless, we are still confidently anticipating that a breakthrough for practical-application PSPEs in LIBs will occur in the near future, according to the rapid emergence of novel synthetic methods.
